# What difference does it make if viruses are strain-, rather than species-specific?

**DOI:** 10.3389/fmicb.2015.00320

**Published:** 2015-04-20

**Authors:** T. Frede Thingstad, Bernadette Pree, Jarl Giske, Selina Våge

**Affiliations:** Department of Biology, Hjort Centre for Marine Ecosystem Dynamics, University of BergenBergen, Norway

**Keywords:** killing-the-winner model, biodiversity control, biodiversity–ecosystem functioning relationships, Weinbauer's Paradox, fractal similarity, microevolution

## Abstract

Theoretical work has suggested an important role of lytic viruses in controlling the diversity of their prokaryotic hosts. Yet, providing strong experimental or observational support (or refutation) for this has proven evasive. Such models have usually assumed “host groups” to correspond to the “species” level, typically delimited by 16S rRNA gene sequence data. Recent model developments take into account the resolution of species into strains with differences in their susceptibility to viral attack. With strains as the host groups, the models will have explicit viral control of abundance at strain level, combined with explicit predator or resource control at community level, but the direct viral control at species level then disappears. Abundance of a species therefore emerges as the combination of how many strains, and at what abundance, this species can establish in competition with other species from a seeding community. We here discuss how species diversification and strain diversification may introduce competitors and defenders, respectively, and that the balance between the two may be a factor in the control of species diversity in mature natural communities. These models can also give a dominance of individuals from strains with high cost of resistance; suggesting that the high proportion of “dormant“ cells among pelagic heterotrophic prokaryotes may reflect their need for expensive defense rather than the lack of suitable growth substrates in their environment.

## Introduction

Classical discussions of diversity-maintaining mechanisms in microbiology had a tendency to focus on competition, with the works of Hutchinson (1961) and Tilman (1977) in phytoplankton ecology as influential examples. With competitive exclusion as the theoretical outcome for species competing for the same limiting substrate, this created a need for additional hypotheses to explain observed microbial biodiversity. With the huge microbial diversity revealed by modern metagenomics (Venter et al., 2004), this gap between observation and theory is now perhaps wider than ever.

The list of mechanisms thought to maintain diversity includes, among others, substrate diversity (Tilman, 1977), environmental variability in time and/or space (Connell, 1979; Beninca et al., 2008; Dini-Andreote et al., 2014; Jankowski et al., 2014; Staley et al., 2014; Uksa et al., 2014), chaotic dynamics (Beninca et al., 2008), and selective loss (Thingstad and Lignell, 1997; Thingstad, 2000), presumably working together in a more or less additive manner in complex natural environments. In the prokaryotic world, the issue of species diversity is further complicated not only by the problem of defining what constitutes a species (Fraser et al., 2009), but also by the fact that strains from the same species contain non-conserved, non-core genes that are sporadically distributed among members of the population (Avrani et al., 2011).

Quite different from a picture of natural communities as assemblages of clonal species populations, this suggests that a prokaryote “species” consists of a set of strains where some traits characterize the set, while other traits vary between strains. Any comparison between model and data then needs to take into account whether the experimental data resolves the genetic information at species or at strain level.

**Figure 1:**
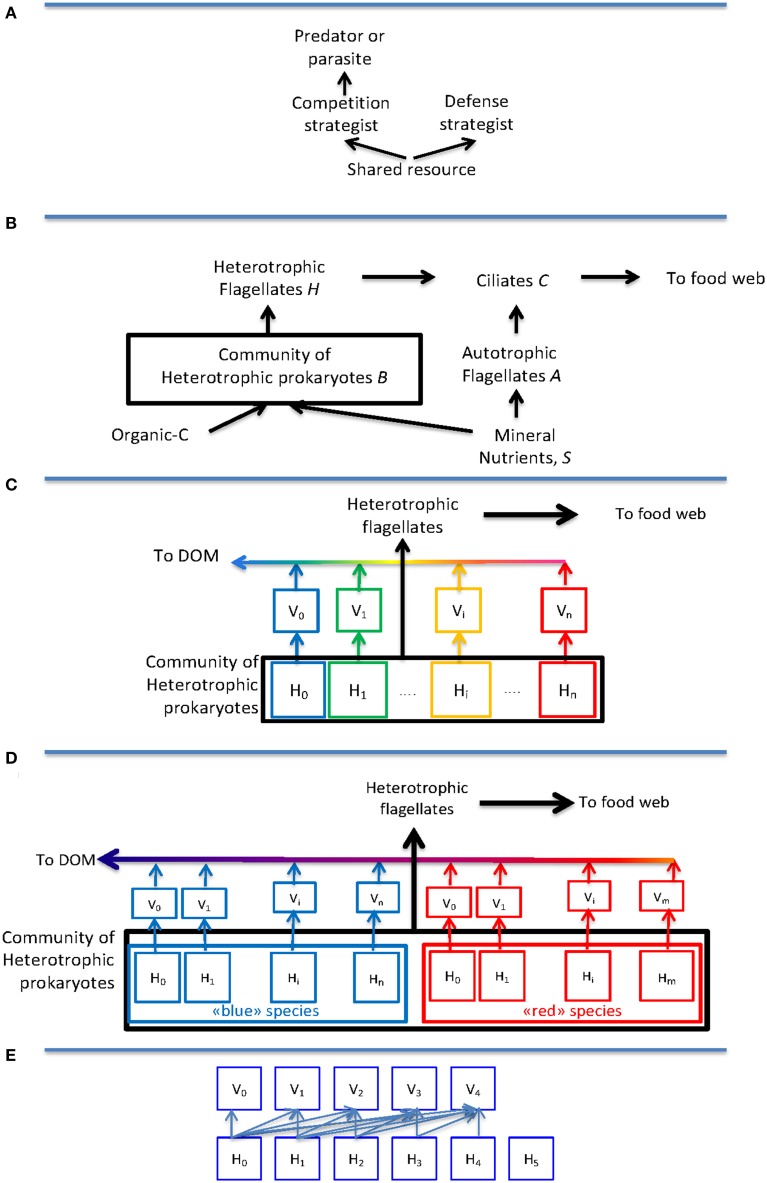
**Idealized models for trophic interactions discussed: (A) The “Killing-the-Winner” structure where abundance of the competition strategist is top-down controlled by a predator or parasite, thereby leaving resources for a resource-limited defense strategist. (B)** An idealized model of the microbial food web based on the principles from **(A)**, illustrating how ciliates influence both biomass and growth conditions of heterotrophic flagellates through their grazing on heterotrophic and autotrophic flagellates, respectively. **(C)** The original one host–one virus model interpreted as host-groups corresponding to species (“blue,” “green,” “yellow,” “red”) and species abundance therefore being top-down controlled. In this model, the application of the KtW principle at the predator-prey creates a transport “up” the food chain, while applying the same principle to viruses sends material “down” to dissolved organic material (DOM). **(D)** Modification of **(C)** by the assumption that host groups correspond to strains belonging to either a “blue” or a “red” species, illustrating how the direct top-down control of abundance disappears at the intermediate level of species. **(E)** Modification of the host-virus interaction from the one host–one virus relationship in **(D)** to a nested structure. This is the structure used in drawing **Figure 2C**.

As a kind of minimum model to describe the effect of top-down control, one can combine size-selective predators with host specific viruses. This creates a two-level model where the same basic “killing-the winner” (KtW) principle (Figure 1A) is applied at both levels: Grazing control of the community size of heterotrophic prokaryotes allows for phytoplankton coexistence with heterotrophic bacteria (Figure 1B), even when bacteria are superior competitors for a shared limiting mineral nutrient (Pengerud et al., 1987). Applying the KtW principle inside the community (Figure 1C) allows competitive host-groups with high growth rate to coexist with defensive host-groups with a low loss to viral lysis, even if this defense comes at the expense of a slower growth rate (Bohannan and Lenski, 2000).

A lot of the experimental and observational work in the field has tried to relate their findings to this KtW model, but clear support (or rebuttal) of the theory has so far been evasive (see e.g., Winter et al., 2010). We recently suggested (Thingstad et al., 2014) that part of this problem might be rooted in the often implicitly made assumption that this model's “host groups” correspond to observational information contained in 16S rRNA gene sequence data. Since such data are more relevant to the species than to a strain level, it means that the KtW model in this case is interpreted with host groups representing species.

Models that incorporate the possibility for viral strain specificity have recently been introduced (Jover et al., 2013; Thingstad et al., 2014). In these, the explicit top-down control on species abundances disappears as illustrated in Figure 1D. This obviously is important for discussions of community “species” composition such as e.g., recent attempts to combine the high abundance of the SAR11 clade with its susceptibility to viral infection (Giovannoni et al., 2013; Våge et al., 2013; Zhao et al., 2013).

In the model of Figure 1D, the abundance of individuals belonging to a species is the sum of individuals belonging to each of its strains, with no other explicit control on species size than the maximum value given by the community size. As a consequence, abundance at the species level becomes a combination of its ability to establish many strains, and the virus-controlled abundance of individuals within each of these strains. Since establishment of a strain and abundance within this strain are functions of its competitive and its defensive abilities, respectively, abundance at species level becomes a combined function of a species' competitive and defensive abilities (Thingstad et al., 2014).

To illustrate the ecological consequences of these models we have arranged the subsequent discussion in sections devoted to a set of questions picked from the list of themes announced for this special issue.

## How does a foreign microbe become a member of the ecosystem?

In a system at steady state, specific growth rate (μ) must equal specific loss rate (δ) for all populations and all subpopulations, so that their net growth rate μ − δ is zero. This system can then be invaded by any new organism type *x*, be it a mutant or an immigrant species, for which μ_*x*_ – δ_*x*_ > 0 when it enters the established community (Symbols summarized in Table 1). The invasion changes the growth conditions in the established community. How numerous the invader will become in the new steady state (if this exists) depends on a new set of equilibrium conditions. This set includes the new μ_*x*_ – δ_*x*_ = 0 condition, while the equivalent condition for now excluded members in the previous state of the community has disappeared. Although, both invasion and final community size for the invader depend on how μ and δ vary with the state of the community, the conditions for invasion and population size are thus different.

**Table 1:** **List of symbols**.

**DESCRIPTION OF HOST SPECIES *x***	
*_x_H_i_*	Abundance of host strain *i* belonging to species *x*
*_x_*μ*_i_*	Specific growth rate for host strain *i* of species *x*. For the graphs in **Figure 2** this is calculated from the Michaelis-Menten relationship: ${}_{x}\mu i\left(S\right)=\frac{{}_{x}\alpha_{S}}{1+\frac{{}_{x}\alpha_{S}}{{}_{x}\mu^{max}{}_{x}v^{i}}}$
*_x_*δ	Specific loss rate host *x*
Competitive abilities of host species *x* influencing the number of strains it can establish	
_*x*_α	Nutrient affinity
_*x*_μ^*max*^	Maximum specific growth rate
_*x*_ν	Fractional reduction in _*x*_μ^*max*^ for each mutational step in the host
Defensive properties of host species *x* influencing the abundance of individuals within strains	
*_x_*β	Adsorbtion coefficient
*_x_*ρ	Parameter 0 < ρ < 1 representing the loss in effective adsorption coefficient for each new host strain added to the arms race
*_x_*σ	Parameter 0 < *σ < 1* representing the fractional loss in effective adsorption coefficient to previous hosts for each new virus strain added to the arms race
**VIRUS PROPERTIES**	
δ*_V_*	Specific loss rate virus
*m*	Burst size virus
**PHYSICAL, CHEMICAL, AND BIOLOGICAL PARAMETERS DEFINING THE ENVIRONMENT AND THE FOOD WEB EFFECTS**	
*D*	Chemostat dilution rate
*S_R_*	Concentration of limiting element in the chemostat reservoir
*S_C_*	Concentration of limiting substrate in the culture
*A*	Population size autotrophic flagellates
*C*	Population size ciliates
*H*	Population size heterotrophic flagellates
*α_A_*	Affinity for uptake of limiting nutrient, autotrophic flagellates
*α_C_*	Clearance rate for ciliate grazing on flagellates
*α_H_*	Clearance rate for heterotrophic flagellates grazing on bacteria
δ*_B_*	Non-viral bacterial loss rate
*Y_H_*	Yield of heterotrophic flagellates on bacterial prey

Two cases are of particular interest for our subsequent discussion, (1) a mutant that can use some unexploited resource in the system and therefore has a large μ_*x*_; and (2) an organism that has few/no enemies in the system or is good in defending itself against those already present, and therefore has a low δ_*x*_.

## How do microbes in ecosystems evolve?

The importance of the somewhat abstract reasoning above can be illustrated by considering the idealized environment of a chemostat, frequently used to study simplified virus-host systems (e.g., Bohannan and Lenski, 1997, 2000; Middelboe et al., 2009; Marston et al., 2012). This simple environment is characterized by two parameters: the dilution rate *D*, and the reservoir concentration *S_R_* of limiting substrate (for simplicity assumed here to be a non-respired substance like e.g., phosphate).

In the traditional theoretical case of a clonal bacterial population, the chemostat will have a resource-controlled population size proportional to *S_R_* and a growth rate equal to the dilution rate: μ(*S_C_*) = *D*. The steady-state culture concentration *S_C_* is thus linked to dilution rate through the growth characteristics of the organism. This clonal community is invadable only by a better competitor, i.e., one that has a higher growth rate at the given *S_C_*. If, however, the established population is infected with a lytic virus, the original susceptible clone becomes virus-controlled and is reduced to a low population level. Part of the available reservoir substrate *S_R_* will then remain unused in the culture, i.e., leading to a high *S_C_*. With a high *S_C_*, this one host-one virus community will be easily invadable by a resistant host mutant, even if the mutation comes at a price (a cost of resistance, COR). With higher *S_C_*, the original parent strain will now grow faster than *D*, it can therefore compensate for both dilution and viral loss and can therefore remain in the culture at a low, virus-controlled, density in coexistence with the now abundant, resource-controlled, immune mutant. The abundant mutant population represents a resource exploitable for a mutant of the virus. Establishment of such a mutant virus gives a new situation with two low-abundant host strains and high *S_C_*, again representing a resource for a new host mutant, and so on. This evolutionary sequence is thus characterized by a “remaining resource” that alternates in form between free limiting nutrients facilitating a successful mutation in the host, and an immune host strain facilitating successful mutation in the virus. This is a model of the “Red Queen” dynamics observed in chemostat experiments of this type (Little, 2002; Martiny et al., 2014). Importantly, the remaining free resource is diminishing for each turn of the arms race until the final resistant host strain has a population size too small to carry a new virus mutant. In a more generalized analysis, Härter et al. (2014) describe such maturation processes as “narrowing staircase(s) of coexistence.” Since the size of the “free resource” would affect the probability that a random mutation is successful, this could explain why antagonistic evolution is fast, but would also suggest that the rate diminishes as the process approaches maturation. The mature state in this arms race model is characterized by many virus-susceptible strains, while the initial stages are dominated by a resistant strain and have a small virus population. Interestingly, these are the two situations forming what has been termed Weinbauer's Paradox (Weinbauer, 2004), where the question is why the few virus—many resistant hosts situation is observed when trying to construct simple host-virus systems in the laboratory, while natural systems are characterized by high viral numbers and many susceptible hosts. In the arms race model above, this apparent paradox is explained simply as the differences between an early and a late stage in the maturation process of strain diversification (Thingstad et al., 2014). How long time is needed for such strain diversifications to mature, and to what extent the speed of the arms race diminishes with the decrease in remaining resource, remain as open questions.

In their analysis of phage-bacteria infection networks, Weitz et al. (2013) defined four basic types: random, one-to-one, nested, and modular. The arms race model outlined for the chemostat example above leads to the nested type with communities that are connected as illustrated in Figure 1E, corresponding to an upper-triangular interaction matrix (Weitz et al., 2013; Thingstad et al., 2014). Such upper-triangular interaction matrices have been found in analysis of published host-virus networks (Flores et al., 2011; Beckett and Williams, 2013), providing some evidence that the highly idealized chemostat analysis used here may bear some relevance to natural systems. The validity of such an extrapolation to natural systems is not self-evident since each species does not evolve independently in a complex community: There, the free resource will be available to all competing species and a high-abundant immune host strain would be available to mutants from many different viruses, opening for models that could lead to more complex structures. With the assumption that no viruses infect hosts belonging to more than one species, the interaction matrix for the community will consist of upper-triangular sub-matrices, one for each species, arranged along the diagonal (Thingstad et al., 2014), very similar to the modularity found in an existing dataset (Beckett and Williams, 2013). The potentially more complex interactions of multispecies communities would be represented by non-zero elements in the void spaces of such a community interaction matrix. A search for the consequences of such complications would require a systematic approach to how to fill in these void spaces and has not been attempted here.

## How is microbial community composition determined?

### The richness component

**Figure 2:**
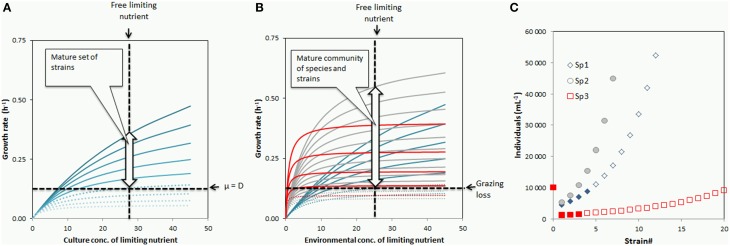
**Growth rate curves illustrating the case with (A) one species diversifying into virus-controlled strains in a chemostat, and (B) a simplified case with three species (“gray,” “blue,” and “red”; defined in Table 2) embedded in the simplified food web of Figure 1B**. Vertical and horizontal lines (black, broken) represent steady state concentration of limiting nutrient and non-specific (non-viral) loss, respectively. The horizontal line thus represents dilution loss in **(A)**, and grazing loss from heterotrophic flagellates in **(B)**. Strains able to establish (indicated by black arrow) are those that have a growth curve crossing the vertical line above the horizontal line. Strains with too high cost of resistance to establish in the chemostat are indicated by dotted growth curves in **(A)**. **(C)** Individuals per strain for the “gray,” “blue,” and “red” species defined in Table 2.

In the chemostat example discussed above, the mechanism generating strain richness in the mature community can be visualized by the set of growth curves for the strains shown in Figure 2A, where a cost of resistance (COR) is assumed in the form of a reduction in maximum growth rate for each successive mutation. The community will consist of all strains for which μ_*i*_(*S_C_*) ≥ D where *D* and *S_C_* are represented by the horizontal and the vertical lines in Figure 2A, respectively. As discussed above, the culture concentration *S_C_* will increase as the arms race proceeds, illustrated by a shifting of the vertical line in Figure 2A one step to the right for each turn of the arms race, adding subsequently less competitive strains to the established host community. This will continue until either (1) the COR has become too large for a new mutant to compensate for dilution, or (2) the community size reaches the carrying capacity defined by the reservoir concentration *S_R_*. In this chemostat model, viral loss is the only mechanism compensating for growth that exceeds dilution loss. The amount of viruses in the mature situation is thus the amount needed to give each strain *i* a loss rate by viral lysis equal to μ_*i*_(*S_C_*)−D.

**Table 2:** **Numerical values used to draw the growth curves and the abundance per strain for the three species in Figures 2B,C**.

	**Competitive traits**			**Defensive traits**					
	**α nM−P^−1^ h^−1^**	**μ*^max^* h^−1^**	**ν**	***H*_0_ mL^−1^**	**σ**	**ρ**	**Trade-off index**	**Species abundance mL^−1^**	**% of community abundance**
Sp#1≪blue≫	0.2	1	0.7	1 10^4^	0.8	0.8	1.5	3.6 10^4^	10
Sp#2≪gray≫	0.1	0.7	0.85	1 10^4^	0.9	0.7	0.35	3.0 10^5^	86
Sp#3 ≪red≫	0.5	0.4	0.7	1 10^4^	0.99	0.9	1.2	1.4 10^4^	4
Community abundance:								3.5 10^5^	

*Trade-off index calculated as described in Thingstad et al. (2014)*.

The main viral loss is thus associated with the fastest growing strains, which, as we will see later, are not the same as the dominant strains.

This graphical representation is easily extrapolated to a multi-species situation with grazing control of community size. This is illustrated for a 3-species case in Figure 2B, where the horizontal line now represents a non-selective grazing loss.

Application of the KtW principle at the food web level allows for models that contain a shift between mineral nutrient limited and carbon limited growth of the heterotrophic prokaryotes (Thingstad and Pengerud, 1985). The nature of the limiting element represented on the x-axis of Figure 2B may therefore change with the environmental conditions with at least organic carbon, nitrogen, phosphorous and iron as expected possibilities. If trade-offs at the species level are mainly between the three parameters defining the topology of Figure 2B (μ^*max*^, α, and ν) one could conceive a situation where the topology of Figure 2B, and therefore the community structure, is invariant to such qualitative shifts in limitation. If, however, there is trade-off between specializations so that e.g., a good competitor under C-limitation is a poor competitor under P-limitation, the growth curves would reshuffle as the type of limitation changes, thereby inducing shifts in species/strain composition.

### The evenness component

Figure 2B does not provide an answer to the evenness aspect of strain diversity since it does not constrain the abundance of individuals within each established strain. This abundance is controlled by the virus host-interactions, i.e., by the structure of the interaction matrix. For the nested structure in Figure 1E, the abundance of the undefended parent strain of species *x* is given as Thingstad et al. (2014):

(1a) $${}_{x}H_{0}=\frac{\delta_{V}+D}{(m-1)\beta }.$$

While abundance of subsequent mutant strains are given by:

(1b) $${}_{x}H_{i}={\;}_{x}H_{0}\frac{1-\sigma \rho }{\rho^{i}},\;i≧1.$$

Equation 1a arises from the equilibrium condition for the first virus which only can infect the parent strain. Abundance of the parent strain thus depends on the decay rate of the virus (δ_*V*_), the burst size (*m*), and the effective adsorption rate (β) between the undefended parent strain and the original virus. For the subsequent strains, this is modified by the two factors ρ, that represents a fractional loss in β for each new host mutant, and σ which represents a fractional loss in infectivity for previous hosts for each mutational step in the virus. The model thus contains mechanism where there is a cost in the form of reduced infectivity for viruses having a broad host range (Symbols summarized in Table 1, see Thingstad et al., 2014 for further details). Abundance within strains is thus related to the species' defensive properties, in particular the parameter 0 < ρ < 1, where values of ρ << 1 give a very rapid increase in strain size for large *i* (Equation 1b). Since all the parameters β, *m*, δ_*V*_, ρ, and σ may vary between host species and between viruses, both the abundance of the parent host strain (Equation 1a) and the modification of this with subsequent strains (Equation 1b) will vary between species. The set of parameters defining a species can thus be divided into two sets: one (α, μ^*max*^, and ν, see Table 1) that describes the species' competitive properties and determining how many strains it can establish, and another (β, ρ, and σ) that defines its defensive properties and, together with the properties of its virus (*m*, δ_*V*_), determines the abundance within strains. Since abundance at species level is the sum over established strains, this will depend on both the competitive and the defensive properties of the species.

Such a combination of high competitive with high defensive abilities requires a low trade-off between the two. With this insight, the question of SAR11 abundance should probably not be focused on whether this is a competition or a defense strategist, but rather whether there is something particular in the lifestyle of this organism that leads to a low trade-off between the two traits (Thingstad et al., 2014). This leads to the intriguing question of whether there is any link between the minimalistic genome design of SAR11 (Giovannoni et al., 2013, 2014) and the potential for a low trade-off between competition and defense. The answer to this is not immediately obvious from the analysis done here.

In the arms race scenario above, a host mutant needs to develop resistance to a virus community that already has co-evolved with the established host community, and it has to do so without losing too much in competitive ability. This could seem like a comparably more difficult task than the “rabbits in Australia” analogy with invasion of a foreign species not recognized by the established virus community and therefore initially not necessarily handicapped by high COR. Although viral attack on invading organisms has been demonstrated experimentally (Sano et al., 2004), such mature communities could thus theoretically seem prone to invasion. Including invasion in the models would create a new maturation process where species richness increases at a rate driven by a combination of the invasion rate and the distribution of competitive abilities within the seeding community. The result would be an interaction between processes occurring at, at least, five characteristic time scales: the population dynamics (linked to the μ and δ functions), the time scale of the host-virus arms races driving strain diversification, the time scale of the species invasion process, and the time scale of environmental disturbances disrupting these maturation processes. On top of this comes the presumably longer time scale of evolution of new species, changing the properties of the seeding community. An important question is whether there is a theoretical balance between these processes, leading to a steady state combination of species and strains somewhere between the two extremes of (1) a single species with many strains on one side and (2) many species, each with only one strain, on the other. The problem is easiest to illustrate in the chemostat example (Figure 2A) where the position of the horizontal line is fixed by the value of *D*. As discussed above, the maturation in strain diversification that establishes new defense specialists will drive the vertical line representing *S* to the right. A successful invasion (or evolution) of a new species will establish a new competition strategist competing better than the last established strain in the existing community. This would exchange previously established strains with low competitive ability (high COR) for a strain from the more competitive newcomer, and thereby drive the vertical line to the left. With species and strain diversification introducing new competition and new defense strategists as assumed here, one therefore gets two opposing processes, suggesting that there is a theoretical balancing point, and therefore a theoretical steady state somewhere between the two extremes outlined above.

This gets a bit more complicated in Figure 2B where the position of the horizontal line is a dynamic variable coupled to grazing loss. Using the simple food web model in Figure 1B, the state with mineral nutrient limited bacteria is, however, still relatively transparent: Assuming steady state in the mineral nutrients (*S*)—autotrophic flagellates (*A*)—ciliates (*C*)—food chain, the autotrophic flagellates must grow as fast as they are grazed. With the simplifying assumption that food intake is proportional to food concentration, the growth = loss condition for autotrophic flagellates becomes α_*A*_*SA* = α_*C*_*AC*, or:

(3a) $$S=\frac{\alpha_{C}}{\alpha_{A}}C.$$

The position of the vertical line (*S*) is therefore now constrained by ciliate abundance (*C*) and not directly controlled by introduction of new species or strains as in the chemostat example above. Instead, it is now the position of the horizontal line that becomes a function of strains and species diversity. Using a similar steady state argument for heterotrophic flagellates, the size of the bacterial community becomes proportional to ciliate abundance:

(3b) $$B=\frac{\alpha_{C}}{Y_{H}\alpha_{H}}C.$$

in a non-mature community, the strain diversification process will thus fill the community with successively less competitive strains, thereby driving the horizontal line downwards until the community size given by Equation (2b) is reached. A new species, sufficiently competitive to invade and initially without viral loss, will outcompete existing strains and thereby drive the horizontal line upwards. Since the horizontal line represents the bacterial loss rate to grazing:

(3c) $$\delta_{B}=\alpha_{H}H,$$

its position reflects the amount (*H*) of bacterial grazers. The strain diversification process thus drives the system toward a smaller population of heterotrophic flagellates and reduced food chain efficiency, thus favoring the viral shunt of bacterial production back to dissolved material. The invasion (or mutation) of new, more competitive species drives the system in the other direction toward less viruses, a smaller virus-bacteria ratio and a more efficient food chain. As the two diversification processes drive the system in opposite directions, this raises the question of whether there is an equilibrium point where the two processes balance?

Equations (2b) and (2c) link the abundance of heterotrophic flagellates (*H*) to the abundance of ciliates (*C*), through the internal population structure of the bacterial community. Stated differently, they suggest a theory where the internal diversity of the bacterial community and the structure of the predator food chain are intimately connected. The analysis above thus suggests a mechanistic basis for a biodiversity—ecosystem functioning (BEF) theory for the pelagic microbial community. It is noteworthy that these relationships were derived without any assumption of host diversity being linked to substrate specialization in the hosts, and are therefore quite different from arguments where a high biodiversity is needed to perform a large set of required biochemical reactions. The food web framework used for this analysis (Figure 1B) may appear unrealistically simple, but its ability to capture essential aspects of mesocosm perturbation experiments recently been demonstrated (Larsen et al., 2015). This includes the demonstration of a central controlling role for ciliates as assumed in the arguments for Equations (2b) and (2c).

The fundamental set of trophic interactions driving the arms races discussed here is summarized in the idealized structure of the KtW principle (Figure 1A). With this basic mechanism being the same at the predator-prey as at the host-parasite level, similar lines of argument can be applied for food web evolution as those used in the above discussion of community composition. The difference in time scale is, however, vast: Whereas the modern pelagic food web can be seen as the present (mature?) state of a 3.5 10^9^ year old arms race with a characteristic time scale in the order of 0.5 10^9^ years (Thingstad et al., 2010) between major new inventions in weapon technology; Red Queen dynamics of prokaryote hosts and viruses seem to occur more over time scale of days to weeks (Martiny et al., 2014).

In such a geological perspective, there may have been strong couplings between these arms races and the global climate systems as for example the rapid evolution of eukaryotic forms in the Neoproterozoic has been suggested to affect the biological pump and therefore may have been a driving force for rather than a response to extreme climatic fluctuations in this period (Lenton et al., 2014).

## How is the physiological state of microbes affected by the environment and other microbes?

The viral top-down control in this model does not only allow competitively inferior microbes to become members of the ecosystem, it rather suggests that the prokaryote community should be dominated by slow-growing, high-defensive strains. This opens for a possible “sieged city” re-interpretation of the old “dormant or dead” (Zweifel and Hagstrom, 1995; Jones and Lennon, 2010) debate in marine microbiology: The dominance of low-active cells found in aquatic environments is perhaps not a sign of starvation caused by a lack of suitable substrates in their environment; it may be more because they have “shut the gates to keep the enemy out.”

## How do (apparently) inferior microbes persist in the ecosystem?

The KtW structure (Figure 1A) was originally designed to provide an answer to this question at the food web level, where the discovery of systems with apparent persistent phosphorous-limitation of bacteria (Pengerud et al., 1987; Vadstein et al., 1988; Cotner et al., 1997) raised the question of why their supposedly inferior phytoplankton competitors were not outcompeted to a level where bacteria would become C-limited. The same basic theory has also been applied to the fish-jellyfish dichotomy at higher levels in the pelagic food chain (Haraldsson et al., 2012). It is also a basic mechanism in size-structured plankton models where the intense grazing and rapid response of flagellates explains the (relatively) constant 10^6^ mL^−1^ abundance of prokaryotes as predator (top-down) control (Azam et al., 1983). Adding nutrients to the system allows large-celled phytoplankton species to establish that are less competitive but also less grazed (resource controlled). Such models thus produce a gradient from small-celled, top-down controlled, “competition strategist” species dominating in oligotrophic regions to large-celled, resource controlled, “defense strategist” phytoplankton dominating nutrient rich upwelling areas (e.g., Ward et al., 2012). As a principle recognizable at many (all?) trophic levels, the KtW structure of Figure 1A generates self-similarity in pelagic ecosystems (Thingstad et al., 2010). This provides at least an analogy to fractal systems where complexity can be generated through the repetition of a simple, scale-independent, rule (Mandelbrot, 1982).

## How are microbes distributed in their ecosystems?

The models discussed above use the simplifying assumptions of a stable steady-state in an environment homogenous in time and space. This is an approximation that should be treated with caution since it means that some important aspects of host-virus interactions are lost. There are at least two relevant examples of microbial competitive and defensive behavior that seem to work only if an evolutionary adaptation to micro scale patchiness in aquatic environments is invoked: motility and altruistic suicide.

Motility is usually argued to have little effect on bacterial nutrient acquisition in homogenous environments because of the high efficiency of molecular diffusion at the 1 μm—scale (Jumars et al., 1993). Motility is thus believed only to be of any advantage in patchy environments. Since the diffusivity of viruses is about 2 orders of magnitude lower (0.2–3.10^−7^ cm^2^s^−1^) (Murray and Jackson, 1992) than that of small nutrient molecules (~10^−5^ cm^2^s^−1^), one can turn this around to argue that the collision frequency with viruses should increase significantly with motility. One should thus expect to find motile bacteria only in patchy environments (Blackburn et al., 1997). One can speculate whether this means that the fraction of motile bacteria may be used as a proxy for environmental patchiness, and also whether this is linked to the COR since the foraging gain from swimming must outweigh the COR associated with the increased defense needed by motile hosts.

A somewhat similar argument is linked to the enigma of altruistic suicide, where some species induce apoptosis when infected (Ackermann et al., 2008; Blower et al., 2012). If this kills the host before new viruses are released, it would protect the neighbors from infection. From an evolutionary perspective, this poses the problem that altruistic suicide may not seem like an evolutionary stable strategy since the genes of the altruist are obviously not passed on to any offspring. If, however, the neighbors are close relatives, the patch becomes a kind of super-organism consisting of cells having the same genes, and altruistic suicide becomes evolutionarily stable (Smith et al., 2010), as has also been shown experimentally (Berngruber et al., 2013). With lytic events creating centers of release for both viruses and DOM, viral lysis is in itself a patchiness-generating mechanism with an interesting geometry consisting of two concentric spheres of food and danger expanding at different rates.

## Concluding comments

The consequences of applying the KtW principles at strain rather than species level are both practical and conceptual. The practical part is that the models then no longer constrain species diversity in the same manner as in the case of pure top-down control of species size, with direct consequences for model comparison with e.g., 16S rRNA gene sequence data. A main conceptual consequence, at least in the model of Thingstad et al. (2014), is the separation of the two aspects of abundance control: The number of strains being controlled by the competitive properties of the host, and the number of individuals per strain by the host's defensive abilities and the host-virus interactions. This separation may seem like a relatively robust feature transferable to models with more complicated interaction matrices. The structure of the interaction matrix would then primarily affect the abundance within strains feature of the system.

The Thingstad et al. (2014) model also involves a seeding community. While this can be approached from a descriptive side with a laborious description of the competitive and defensive properties of “all” existing host species; the intriguing challenge is to try to understand the trade-offs between traits in this population. One can visualize this problem in an n-dimensional strategy space formed by the n relevant life-history traits of hosts and viruses. Trade-offs represent correlations in this n-dimensional space and would confine the subset of feasible strategies. With a slight rephrasing of Baas Becking and Beijerinck's famous statement (de Wit and Bouvier, 2006), this would give models where “The feasible is everywhere, the environment selects.”

### Conflict of interest statement

The authors declare that the research was conducted in the absence of any commercial or financial relationships that could be construed as a potential conflict of interest.

## References

[B1] Ackermann M. Stecher B. Freed N. E. Songhet P. Hardt W.-D. Doebeli M. (2008). Self-destructive cooperation mediated by phenotypic noise. Nature 454, 987–990. 10.1038/nature07067 18719588

[B2] Avrani S. Wurtzel O. Sharon I. Sorek R. Lindell D. (2011). Genomic island variability facilitates *Prochlorococcus*-virus coexistence. Nature 474, 604–608. 10.1038/nature10172 21720364

[B3] Azam F. Fenchel T. Field J. G. Gray J. S. Meyer-Reil L. A. Thingstad T. F. (1983). The ecological role of water-column microbes in the sea. Mar. Ecol. Prog. Ser. 10, 257–263 10.3354/meps010257

[B4] Beckett S. J. Williams H. T. P. (2013). Coevolutionary diversification creates nested-modular structure in phage-bacteria interaction networks. Interface Focus 3:20130033. 10.1098/rsfs.2013.0033 24516719 PMC3915849

[B5] Beninca E. Huisman J. Heerkloss R. Johnk K. D. Branco P. Van Nes E. H.. (2008). Chaos in a long-term experiment with a plankton community. Nature 451, 822–827. 10.1038/nature06512 18273017

[B6] Berngruber T. W. Lion S. Gandon S. (2013). Evolution of suicide as a defence strategy against pathogens in a spatially structured environment. Ecol. Lett. 16, 446–453. 10.1111/ele.12064 23331662

[B7] Blackburn N. Azam F. Hagstrom A. (1997). Spatially explicit simulations of a microbial food web. Limnol. Oceanogr. 42, 613–622 10.4319/lo.1997.42.4.0613

[B8] Blower T. R. Evans T. J. Przybilski R. Fineran P. C. Salmond G. P. C. (2012). Viral evasion of a bacterial suicide system by RNA-based molecular mimicry enables infectious altruism. PLoS Genet. 8:e1003023. 10.1371/journal.pgen.1003023 23109916 PMC3475682

[B9] Bohannan B. J. M. Lenski R. E. (1997). Effect of resource enrichment on a chemostat community of bacteria and bacteriophage. Ecology 78, 2303–2315 10.1890/0012-9658(1997)078[2303:EOREOA]2.0.CO;2

[B10] Bohannan B. J. M. Lenski R. E. (2000). The relative importance of competition and predation varies with productivity in a model community. Am. Nat. 156, 329–340 10.1086/303393 29592139

[B11] Connell J. H. (1979). IIntermediate-disturbance hypothesis. Science 204, 1345–1345. 10.1126/science.204.4399.1345 17813175

[B12] Cotner J. B. Ammerman J. W. Peele E. R. Bentzen E. (1997). Phosphorus-limited bacterioplankton growth in the Sargasso Sea. Aquat. Microb. Ecol. 13, 141–149 10.3354/ame013141

[B13] de Wit R. Bouvier T. (2006). ‘Everything is everywhere, but, the environment selects’; what did Baas Becking and Beijerinck really say? Environ. Microbiol. 8, 755–758. 10.1111/j.1462-2920.2006.01017.x 16584487

[B14] Dini-Andreote F. de Cássia Pereira e Silva M. Triado-Margarit X. Casamayor E. O. van Elsas J. D. Salles J. F. (2014). Dynamics of bacterial community succession in a salt marsh chronosequence: evidences for temporal niche partitioning. ISME J. 8, 1989–2001. 10.1038/ismej.2014.54 24739625 PMC4184019

[B15] Flores C. O. Meyer J. R. Valverde S. Farr L. Weitz J. S. (2011). Statistical structure of host-phage interactions. Proc. Natl. Acad. Sci. U.S.A. 108, E288–E297. 10.1073/pnas.1101595108 21709225 PMC3136311

[B16] Fraser C. Alm E. J. Polz M. F. Spratt B. G. Hanage W. P. (2009). The bacterial species challenge: making sense of genetic and ecological diversity. Science 323, 741–746. 10.1126/science.1159388 19197054

[B17] Giovannoni S. Temperton B. Zhao Y. (2013). SAR11 viruses and defensive host strains reply. Nature 499, E4–E5. 10.1038/nature12388 23887435

[B18] Giovannoni S. J. Thrash J. C. Temperton B. (2014). Implications of streamlining theory for microbial ecology. ISME J. 8, 1553–1565. 10.1038/ismej.2014.60 24739623 PMC4817614

[B19] Haraldsson M. Tonnesson K. Tiselius P. Thingstad T. F. Aksnes D. L. (2012). Relationship between fish and jellyfish as a function of eutrophication and water clarity. Mar. Ecol. Prog. Ser. 471, 73–85 10.3354/meps10036

[B20] Härter J. O. Mitarai N. Sneppen K. (2014). Phage and bacteria support mutual diversity in a narrowing staircase of coexistence. ISME J. 8, 2317–2326. 10.1038/ismej.2014.80 24858781 PMC4992086

[B21] Hutchinson G. E. (1961). The paradox of the plankton. Am. Nat. 95, 137–145 10.1086/282171

[B22] Jankowski K. Schindler D. E. Horner-Devine M. C. (2014). Resource availability and spatial heterogeneity control bacterial community response to nutrient enrichment in lakes. PLoS ONE 9:e8699. 10.1371/journal.pone.0086991 24489823 PMC3904960

[B23] Jones S. E. Lennon J. T. (2010). Dormancy contributes to the maintenance of microbial diversity. Proc. Natl. Acad. Sci. U.S.A. 107, 5881–5886. 10.1073/pnas.0912765107 20231463 PMC2851880

[B24] Jover L. F. Cortez M. H. Weitz J. S. (2013). Mechanisms of multi-strain coexistence in host-phage systems with nested infection networks. J. Theor. Biol. 332, 65–77. 10.1016/j.jtbi.2013.04.011 23608631

[B25] Jumars P. Deming J. Hill P. Karp-Boss L. Yager P. Dade W. (1993). Physical constraints on marine osmotrophy in an optimal foraging contex. Mar. Microb. Food Webs 7, 121–159.

[B26] Larsen A. Egge J. K. Nejstgaard J. C. Di Capua I. Thyrhaug R. Bratbak G. (2015). Contrasting response to nutrient manipulation in Arctic mesocosms are reproduced by a minimum microbial food web model. Limnol. Oceanogr. 60, 360–374 10.1002/lno.10025 PMC4459036 26074626

[B27] Lenton T. M. Boyle R. A. Poulton S. W. Shields-Zhou G. A. Butterfield N. J. (2014). Co-evolution of eukaryotes and ocean oxygenation in the Neoproterozoic era. Nat. Geosci. 7, 257–265 10.1038/ngeo2108

[B28] Little T. J. (2002). The evolutionary significance of parasitism: do parasite-driven genetic dynamics occur *ex silico*? J. Evolut. Biol. 15, 1–9 10.1046/j.1420-9101.2002.00366.x

[B29] Mandelbrot B. B. (1982). The Fractal Geometry of Nature. New York, NY: W.H. Freeman and Company.

[B30] Marston M. F. Pierciey F. J. Jr. Shepard A. Gearin G. Qi J. Yandava C.. (2012). Rapid diversification of coevolving marine Synechococcus and a virus. Proc. Natl. Acad. Sci. U.S.A. 109, 4544–4549. 10.1073/pnas.1120310109 22388749 PMC3311363

[B31] Martiny J. B. H. Riemann L. Marston M. F. Middelboe M. (2014). Antagonistic coevolution of marine planktonic viruses and their hosts. Ann. Rev. Mar. Sci. 6, 393–414. 10.1146/annurev-marine-010213-135108 23987913

[B32] Middelboe M. Holmfeldt K. Riemann L. Nybroe O. Haaber J. (2009). Bacteriophages drive strain diversification in a marine Flavobacterium: implications for phage resistance and physiological properties. Environ. Microbiol. 11, 1971–1982. 10.1111/j.1462-2920.2009.01920.x 19508553

[B33] Murray A. G. Jackson G. A. (1992). Viral dynamics - A model of the effects of size, shape, motion and abundance of single-celled planktonic organisms and other particles. Mar. Ecol. Prog. Ser. 89, 103–116 10.3354/meps089103

[B34] Pengerud B. Skjoldal E. F. Thingstad T. F. (1987). The reciprocal interaction between degradation of glucose and ecosystem structure - studies in mixed chemostat cultures of marine bacteria, algae, and bacterivorous nanoflagellates. Mar. Ecol. Prog. Ser. 35, 111–117 10.3354/meps035111

[B35] Sano E. Carlson S. Wegley L. Rohwer F. (2004). Movement of viruses between biomes. Appl. Environ. Microbiol. 70, 5842–5846. 10.1128/AEM.70.10.5842-5846.2004 15466522 PMC522096

[B36] Smith J. Van Dyken J. D. Zee P. C. (2010). A generalization of Hamilton's rule for the evolution of microbial cooperation. Science 328, 1700–1703. 10.1126/science.1189675 20576891 PMC3097903

[B37] Staley C. Gould T. J. Wang P. Phillips J. Cotner J. B. Sadowsky M. J. (2014). Bacterial community structure is indicative of chemical inputs in the Upper Mississippi River. Front. Microbiol. 5:524. 10.3389/fmicb.2014.00524 25339945 PMC4189419

[B38] Thingstad T. F. (2000). Elements of a theory for the mechanisms controlling abundance, diversity, and biogeochemical role of lytic bacterial viruses in aquatic systems. Limnol. Oceanogr. 45, 1320–1328 10.4319/lo.2000.45.6.1320

[B39] Thingstad T. F. Lignell R. (1997). Theoretical models for the control of bacterial growth rate, abundance, diversity and carbon demand. Aquat. Microb. Ecol. 13, 19–27 10.3354/ame013019

[B40] Thingstad T. F. Pengerud B. (1985). Fate and effect of allochthonous organic material in aquatic microbial ecosystems - an analysis based on chemostat theory. Mar. Ecol. Prog. Ser. 21, 47–62 10.3354/meps021047

[B41] Thingstad T. F. Strand E. Larsen A. (2010). Stepwise building of plankton functional type (PFT) models: a feasible route to complex models? Prog. Oceanogr. 84, 6–15 10.1016/j.pocean.2009.09.001

[B42] Thingstad T. F. Våge S. Storesund J. E. Sandaa R.-A. Giske J. (2014). A theoretical analysis of how strain-specific viruses can control microbial species diversity. Proc. Natl. Acad. Sci. U.S.A. 111, 7813–7818. 10.1073/pnas.1400909111 24825894 PMC4040589

[B43] Tilman D. (1977). Resource competition between planktonic algae - Experimental and theoretical approach. Ecology 58, 338–348 10.2307/1935608

[B44] Uksa M. Fischer D. Welzl G. Kautz T. Koepke U. Schloter M. (2014). Community structure of prokaryotes and their functional potential in subsoils is more affected by spatial heterogeneity than by temporal variations. Soil Biol. Biochem. 75, 197–201 10.1016/j.soilbio.2014.04.018

[B45] Vadstein O. Jensen A. Olsen Y. Reinertsen H. (1988). Growth and phosporus status of limnetic phytoplankton and bacteria. Limnol. Oceanogr. 33, 489–503 10.4319/lo.1988.33.4.0489

[B46] Våge S. Storesund J. E. Thingstad T. F. (2013). SAR11 viruses and defensive host strains. Nature 499, E3–E4. 10.1038/nature12387 23887434

[B47] Venter J. C. Remington K. Heidelberg J. F. Halpern A. L. Rusch D. Eisen J. A.. (2004). Environmental genome shotgun sequencing of the Sargasso Sea. Science 304, 66–74. 10.1126/science.1093857 15001713

[B48] Ward B. A. Dutkiewicz S. Jahn O. Follows M. J. (2012). A size-structured food-web model for the global ocean. Limnol. Oceanogr. 57, 1877–1891 10.4319/lo.2012.57.6.1877

[B49] Weinbauer M. G. (2004). Ecology of prokaryotic viruses. FEMS. Microbiol. Ecol. 28, 127–181. 10.1016/j.femsre.2003.08.001 15109783

[B50] Weitz J. S. Poisot T. Meyer J. R. Flores C. O. Valverde S. Sullivan M. B.. (2013). Phage-bacteria infection networks. Trends Microbiol. 21, 82–91. 10.1016/j.tim.2012.11.003 23245704

[B51] Winter C. Bouvier T. Weinbauer M. G. Thingstad T. F. (2010). Trade-Offs between competition and defense specialists among unicellular planktonic organisms: the “killing the winner” hypothesis revisited. Microbiol. Mol. Biol. Rev. 74, 42–57. 10.1128/MMBR.00034-09 20197498 PMC2832346

[B52] Zhao Y. Temperton B. Thrash J. C. Schwalbach M. S. Vergin K. L. Landry Z. C.. (2013). Abundant SAR11 viruses in the ocean. Nature 494, 357–360. 10.1038/nature11921 23407494

[B53] Zweifel U. L. Hagstrom A. (1995). Total counts of marine bacteria include a large fraction of non-nucleoid-containing bacteria (ghosts). Appl. Environ. Microbiol. 61, 2180–2185. 16535043 10.1128/aem.61.6.2180-2185.1995 PMC1388461

